# Predictors and Mortality for Worsening Left Ventricular Ejection Fraction in Patients With HFpEF

**DOI:** 10.3389/fcvm.2022.820178

**Published:** 2022-02-24

**Authors:** Liling Chen, Zhidong Huang, Xiaoli Zhao, Jingjing Liang, Xiaozhao Lu, Yibo He, Yu Kang, Yun Xie, Jin Liu, Yong Liu, Jin Yang, Weixu Yu, Wanling Deng, Yuxiong Pan, Jin Lu, Yanfang Yang, Xujing Xie, Xiaoxian Qian, Qingbo Xu, Longtian Chen, Kaihong Chen, Shiqun Chen

**Affiliations:** ^1^Department of Cardiology, Longyan First Hospital Affiliated of Fujian Medical University, Longyan, China; ^2^Department of Cardiology, Guangdong Cardiovascular Institute, Guangdong Provincial People's Hospital, Guangdong Academy of Medical Sciences, Guangzhou, China; ^3^Department of Cardiology, Guangdong Provincial Key Laboratory of Coronary Heart Disease Prevention, Guangdong Cardiovascular Institute, Guangdong Provincial People's Hospital, Guangdong Academy of Medical Sciences, Guangzhou, China; ^4^Department of Cardiology, The Third Affiliated Hospital of Sun Yat-sen University, Guangzhou, China; ^5^Department of Cardiology, Shunde Hospital, Southern Medical University, Guangzhou, China; ^6^Department of Cardiology, Maoming People's Hospital, Maoming, China; ^7^Department of Hematology, Longyan First Hospital Affiliated of Fujian Medical University, Longyan, China

**Keywords:** heart failure with preserved ejection fraction, worsening LVEF, mortality, incidence, predictor

## Abstract

**Background:**

Definitions of declined left ventricular ejection fraction (LVEF) vary across studies and research results concerning the association of mortality with declined LVEF are inconsistent. Thus, this study aimed to assess the impact of early worsening LVEF on mortality in patients with heart failure (HF) with preserved ejection fraction (HFpEF) and to establish independent predictors of early worsening LVEF.

**Methods and Results:**

A total of 1,418 consecutive patients with HFpEF with LVEF remeasurement from the Cardiorenal Improvement registry were included in this study. Worsening LVEF was defined as an absolute decline ≥ 5% from baseline LVEF within 3 to 12 months after discharge. The Cox and logistic regression analyses were performed to assess prognostic effects and predictors for worsening LVEF, respectively. Among 1,418 patients with HFpEF, 457 (32.2%) patients exhibited worsening LVEF. During a median follow-up of 3.2 years (interquartile range: 2.3–4.0 years), 92 (6.5%) patients died. Patients with HFpEF with worsening LVEF had higher mortality relative to those with nonworsening LVEF [9.2 vs. 5.2%; adjusted hazard ratio (aHR): 2.18, 95% CI: 1.35–3.52]. In the multivariate binary logistic regression analysis, baseline left ventricular end-diastolic dimension (LVEDD), LVEF, high-density lipoprotein cholesterol (HDL-C), atrial fibrillation (AF), and diabetes mellitus (DM) emerged as predictive factors of worsening LVEF.

**Conclusion:**

This study demonstrated that about one out of three patients with HFpEF experiences worsening LVEF during follow-up, which is associated with 2.2-fold increased mortality. Increased LVEDD and LVEF, low HDL-C levels, AF, and DM were predictors of worsening LVEF. Further studies are needed to prospectively assess the efficacy of early active management on prognosis in patients with HF with worsening LVEF.

**Registration:**

ClinicalTrials.gov, identifier NCT04407936.

**Graphical Abstract:**
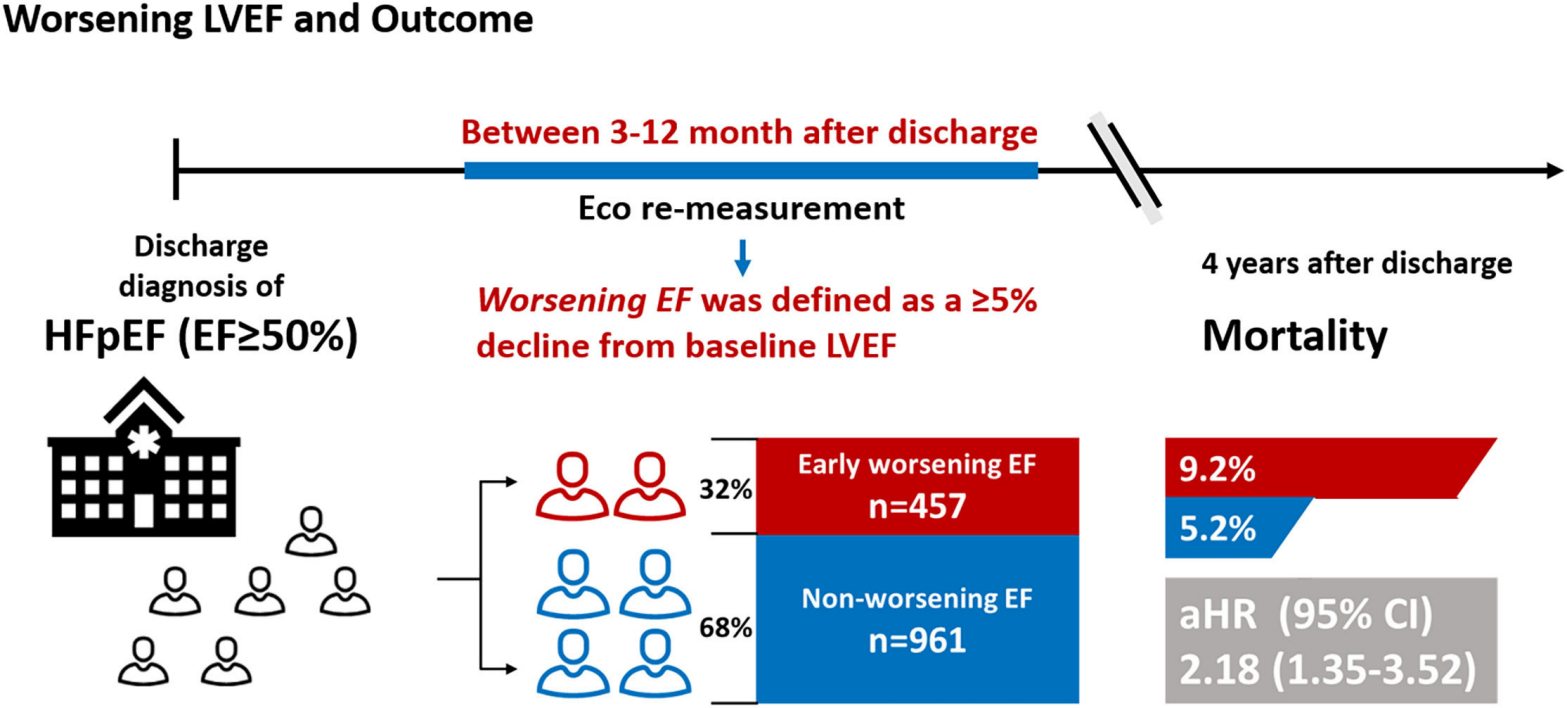
Incidence, predictors, and mortality for worsening LVEF in patients with HFpEF.

## Introduction

Heart failure (HF) with preserved ejection fraction (HFpEF) affects nearly 20 million people worldwide, confers substantial morbidity, and significantly contributes to healthcare costs (1–3). Clinicians may have the preconception that HFpEF has a better prognosis than HF with reduced ejection fraction (HFrEF), but many studies have shown that HFpEF has similar or even higher mortality than HFrEF (4, 5). However, reliable risk stratification systems for patients with HFpEF are still lacking, which make it difficult for physicians to intervene and treat high-risk patients (6). Therefore, identifying high-risk patients with HFpEF based on modifiable and readily accessible clinical risk factors are essential to perform effective management and interventions to reduce mortality.

Recently, modest changes in left ventricular ejection fraction (LVEF) values have been validated as a good prognostic indicator in the general population (7) and may also be effective for patients with HFpEF. Previous studies focusing on declining LVEF represented it as a “transition phenotype” leading to HFrEF, but in fact, only a small proportion of patients with HFpEF show such a decline (8, 9). Furthermore, studies addressing the association of declined LVEF and mortality reported heterogeneous (9, 10). Thus, further studies are needed to verify the impact of LVEF decline on outcomes in patients with HFpEF.

Accordingly, we conducted a retrospective cohort study to assess the impact of early worsening LVEF (defined as an absolute decline ≥ 5% from baseline LVEF between 3 and 12 months after discharge) on mortality in patients with HFpEF and to establish independent predictors of worsening LVEF.

## Methods

### Study Population

**Figure 1:**
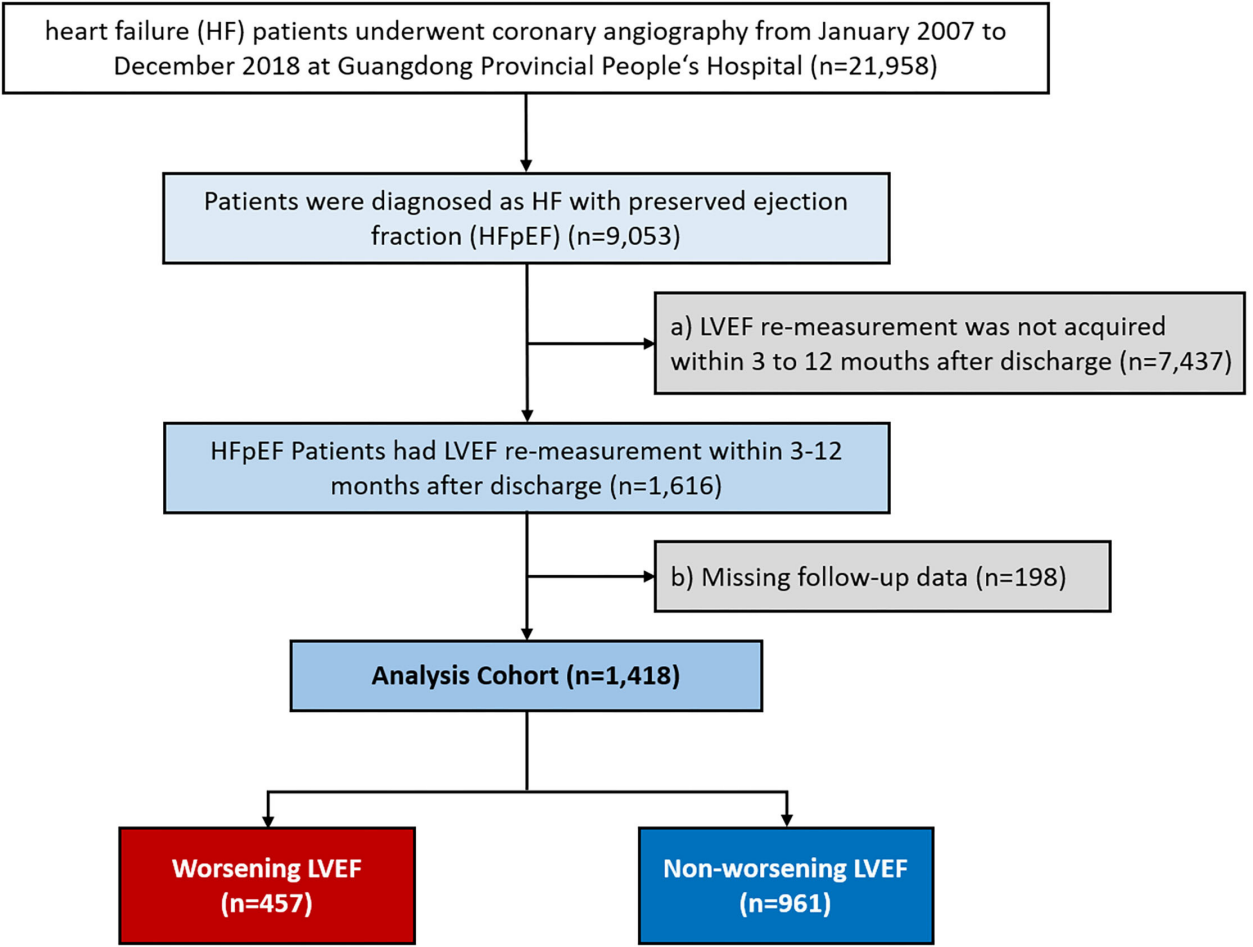
Patient flow diagram.

We consecutively screened 21,958 patients with HF undergoing coronary angiography (CAG) from January 2007 to December 2018 at the Guangdong Provincial People's Hospital-Cardiorenal Improvement Registry (CIN, ClinicalTrials.gov NCT04407936). Of those, 9,053 patients diagnosed with HFpEF (LVEF ≥ 50%) were initially included in this study. HF was diagnosed when meeting one of these criteria: (i) the New York Heart Association (NYHA) class > II or Killip class > I (11); (ii) LVEF < 40%; and (iii) N-terminal pro-B-type natriuretic peptide (NT-proBNP) > 450 pg/ml (age < 50 years), NT-proBNP > 900 pg/ml (age 50 to 75 years), and NT-proBNP > 1,800 pg/ml (age > 75 years) (12, 13). The exclusion criteria were as follows: (a) LVEF remeasurement not acquired within 3 to 12 months after discharge and (b) missing follow-up data (Figure 1). Finally, 1,418 patients were included. The cohort was subsequently divided into the worsening LVEF and nonworsening LVEF groups based on changes in ECG parameters. This study conformed to the principles outlined in the Declaration of Helsinki and was approved by the Guangdong Provincial People's Hospital Institutional Review Board. The Ethics Committee of the hospital waived the requirement of a written informed consent for participation because our review was a retrospective study that involved coded of data reuse.

### Data Collection

We retrieved all the clinical data of the enrolled patients during their first hospitalization from the database of the Guangdong Provincial People's Hospital including demographic characteristics, medical history, procedures, laboratory tests, echocardiography, and use of medications (discharge). Specifically, LVEF values were acquired by quantitative two-dimensional Simpson's biplane method through the end-diastolic and end-systolic apical 4- and 2-chamber views by using transthoracic echocardiography (14). Remeasurement of LVEF was performed over 3–12 months after hospitalization by the same method. When the same patient reported more than two LVEF remeasurements, the last assessments were considered to calculate the change in LVEF.

### Definitions and Outcomes

Worsening LVEF was defined as an absolute decrease ≥ 5% in LVEF from baseline between 3 and 12 months after discharge. The estimated glomerular filtration rate (eGFR) was calculated by the Modification of Diet in Renal Disease (MDRD) formula and chronic kidney disease (CKD) was defined as eGFR < 60 ml/min/1.73 m^2^ (15). Coronary artery disease (CAD), acute myocardial infarction (AMI), hypertension, diabetes mellitus (DM), and atrial fibrillation (AF) were defined by using the International Classification of Diseases-10 (ICD-10) codes. The primary endpoint of this study was all-cause mortality. Data on all-cause mortality and follow-up time were obtained from the Guangdong Provincial Public Security and matched to the electronic Clinical Management System of the Guangdong Provincial People's Hospital records.

### Statistical Analysis

Baseline characteristics are presented as means ± SD or median [interquartile ranges (IQRs)] for continuous variables as appropriate and as number (percentage) for categorical variables. The Student's *t*-test, the Kruskal–Wallis test, and the chi-squared test were used to compare differences between groups as appropriate. Schoenfeld residuals were applied to examine the proportional hazards assumption, the Kaplan–Meier curves were used to present time-to-event data, and log-rank tests were applied to compare survival between groups. The multivariate Cox proportional hazards regression analysis was performed to identify the hazard ratio (HR) and 95% CIs for associations of worsening LVEF with all-cause mortality. To determine the predictors of worsening LVEF, candidate variables were selected based on clinical plausibility and significance on the univariate analyses (*P* < 0.3) and then entered into the multivariate binary logistic regression model by using the backward Wald stepwise regression method (**Table 3**). A two-sided *P* < 0.05 indicated significance for all the analyses. All the statistical analyses were performed by using R software (version 4.0.3).

## Results

### Baseline Characteristics

A total of 1,418 patients with HFpEF were included, among which 457 (32.2%) patients with HFpEF experienced worsening LVEF (mean age, 60.9 ± 10.3 years; 38.29% female) and 961 patients with HFpEF did not experience worsening LVEF (mean age, 60.0 ± 10.1 years; 35.59% female). The worsening LVEF group had higher baseline LVEF, larger left ventricular end-diastolic dimension (LVEDD), and lower re-evaluated LVEF than the nonworsening LVEF group (*P* < 0.05 for all the parameters). There were no significant differences between the groups for all the comorbidities, medications at discharge, and other laboratory examinations (Table 1).

**Table 1:** Baseline characteristics of patients with HFpEF with and without worsening LVEF.

**Characteristics**	**Non-worsening LVEF**	**Worsening LVEF**	***p*-value**
	***N* = 961**	***N* = 457**	
Age, years	60.0 ± 10.1	60.9 ± 10.3	0.145
Female, n (%)	342 (35.6)	175 (38.3)	0.352
**Medical history**
AMI, n (%)	128 (13.3)	63 (13.8)	0.875
CAD, n (%)	343 (35.7)	181 (39.6)	0.171
Hypertension, n (%)	315 (32.8)	165 (36.1)	0.239
DM, n (%)	142 (14.8)	86 (18.8)	0.063
CKD, n (%)	212 (22.1)	104 (22.8)	0.821
AF, n (%)	380 (39.5)	200 (43.8)	0.146
Stroke, n (%)	64 (6.7)	30 (6.6)	>0.99
Cancer, n (%)	13 (1.4)	6 (1.3)	>0.99
COPD, n (%)	7 (0.7)	3 (0.7)	>0.99
Pre-AMI, n (%)	23 (2.4)	7 (1.5)	0.392
Pre-PCI, n (%)	26 (2.8)	19 (4.2)	0.195
In-hospital dialysis, n (%)	28 (2.9)	15 (3.3)	0.832
PCI, n (%)	230 (23.9)	110 (24.1)	>0.99
**Echocardiography**
LVEF, %	60.91 ± 5.54	65.09 ± 7.23	<0.001
Re-measurement LVEF, %	64.56 ± 5.58	53.47 ± 10.49	<0.001
LVEDD, mm	49.03 ± 8.01	50.61 ± 8.50	0.001
LVESD, mm	31.95 ± 6.60	32.06 ± 7.03	0.782
**Laboratory findings**
NT-proBNP, pg/ml	1359.00 [933.60, 2325.00]	1502.00 [985.25, 2507.50]	0.138
eGFR, mL/min/1.73m2	73.77 ± 25.91	73.49 ± 26.22	0.862
LDL-C, mmol/L	2.80 [2.30, 3.44]	2.93 [2.38, 3.44]	0.183
HDL-C, mmol/L	1.02 [0.86, 1.24]	0.99 [0.82, 1.16]	0.016
HbA1c, %	6.14 ± 1.10	6.15 ± 1.03	0.918
ALB, g/L	36.48 ± 4.27	36.38 ± 4.50	0.702
HS-CRP, mg/L	5.02 [1.43, 13.27]	4.14 [1.29, 13.05]	0.671
**Treatment**
B-blocker, n (%)	525 (55.4)	263 (58.1)	0.386
Statins, n (%)	329 (34.7)	169 (37.3)	0.380
Aspirin, n (%)	300 (31.7)	155 (34.2)	0.375
Spirolactone, n (%)	657 (69.4)	323 (71.3)	0.501
CCB, n (%)	140 (14.8)	63 (13.9)	0.723
ACEI/ARB, n (%)	185 (19.5)	96 (21.2)	0.514
Diuretics, n (%)	697 (73.6)	341 (75.3)	0.546

*Data represented as mean ± SD, median [interquartile ranges (IQRs)], and number (%) as appropriate*.

*HFpEF, heart failure with preserved ejection fraction; LVEF, left ventricular ejection fraction; AMI, acute myocardial infarction; CAD, coronary artery disease; DM, diabetes mellitus; CKD, chronic kidney disease; AF, atrial fibrillation; COPD, chronic obstructive pulmonary disease; PCI, percutaneous coronary intervention; eGFR, estimated glomerular filtration rate; LDL-C, low-density lipoprotein cholesterol; HDL-C, high-density lipoprotein cholesterol; HbA1c, hemoglobin A1c; ALB, albumin; HS-CRP, hypersensitive C-reactive proptein; NT-proBNP, N-terminal pro-B-type natriuretic peptide; LVEDD, left ventricular end-diastolic dimension; LVESD, left ventricular end-systolic dimension; LVEF, left ventricle ejection fraction; CCB, calcium channel blocker; ACEI, angiotension-converting enzyme inhibitor; ARB, angiotension receptor blocker*.

### Outcomes

**Figure 2:**
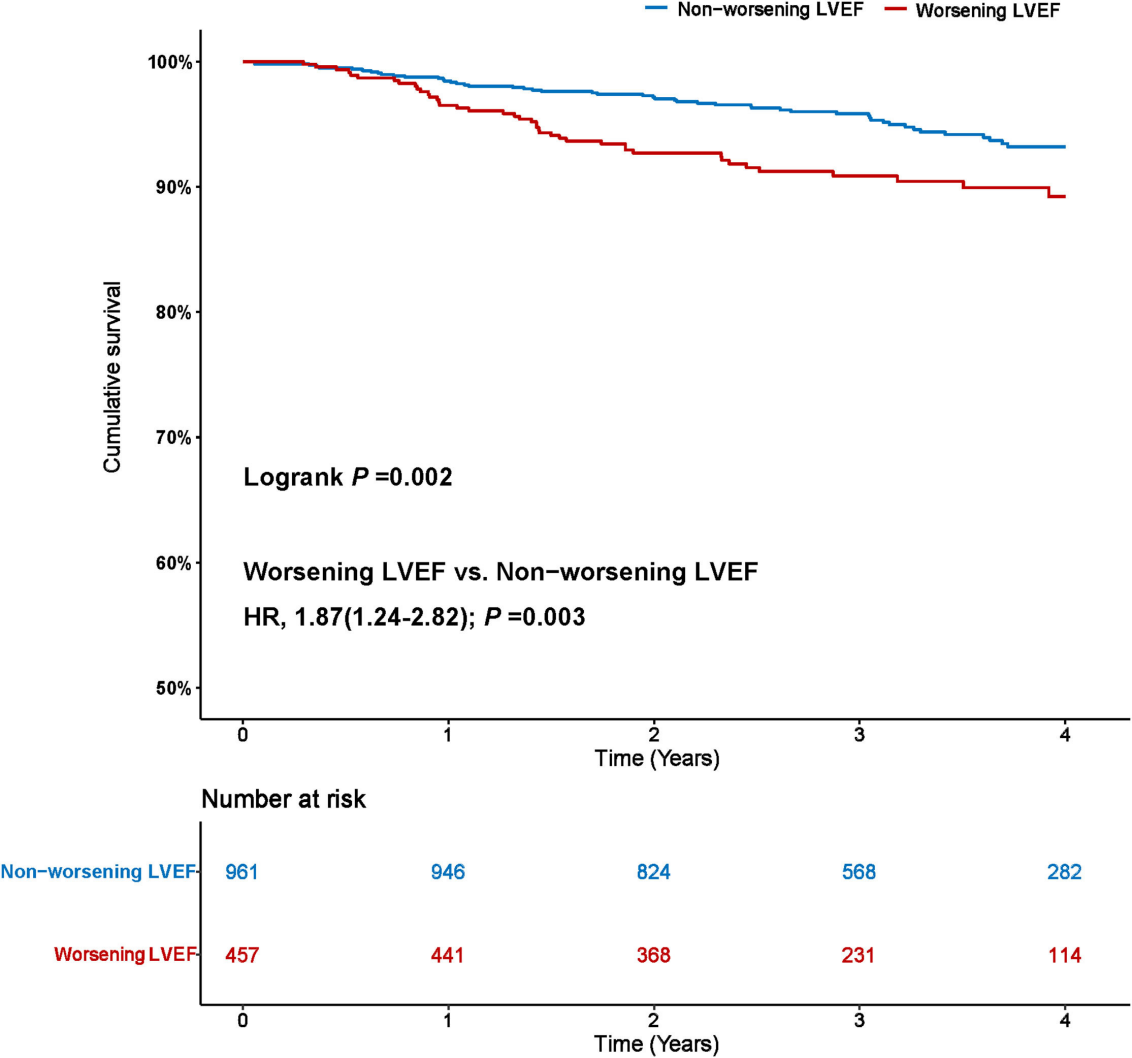
Worsening left ventricular ejection fraction (LVEF) and risk of mortality. The Kaplan–Meier curves for all-cause mortality in patients with heart failure with preserved ejection fraction (HFpEF) with and without worsening LVEF.

**Figure 3:**
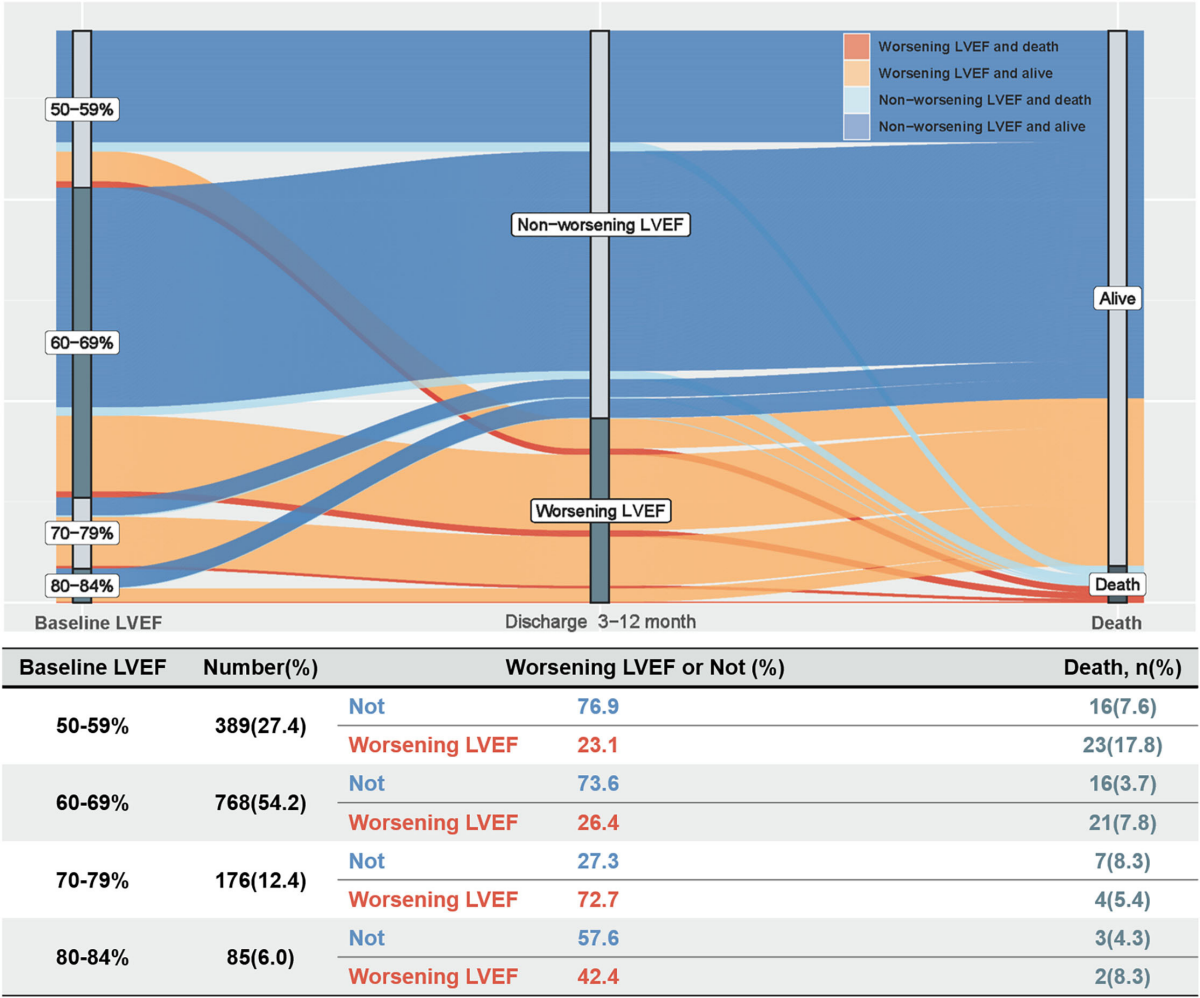
The trajectory of worsening LVEF and eventual mortality in patients with HFpEF.

During a median follow-up of 3.2 years (IQR: 2.3–4.0), 92 (6.5%) patients died. The Kaplan–Meier curve depicting mortality rates for patients with or without worsening LVEF is shown in Figure 2. Worsening LVEF occurred predominantly in patients with an LVEF of 60 to 84% at baseline. In general, patients with HFpEF who developed worsening LVEF had a relatively higher mortality (Figure 3). Additional measurements and the trajectory of worsening LVEF and eventual mortality among patients with HFpEF with different LVEF categories at baseline are given in Figure 3. After controlling confounders, the worsening LVEF group was associated with an increased risk of mortality compared to patients with nonworsening LVEF (9.2 vs. 5.2%, respectively; adjusted HR: 2.18, 95% CI: 1.35–3.52; Table 2).

**Table 2:** The Cox regression analysis with risk factors for all-cause mortality in patients with HFpEF.

**Worsening LVEF *vs*. Non- worsening LVEF**	**HR (95% CI)**	***P*-value**
Model^a^	1.79 (1.19–2.70)	0.006
Model^b^	2.12 (1.32–3.39)	0.002
Model^c^	2.18 (1.35–3.52)	0.001

*Model^a^: Adjustment for demographics (age and sex)*.

*Model^b^: Adjustment for demographics (age and sex), left ventricular ejection fraction, N-terminal pro-B-type natriuretic peptide, high-density lipoprotein cholesterol, estimated glomerular filtration rate, coronary artery disease, acute myocardial infarction, percutaneous coronary intervention, atrial fibrillation, diabetes mellitus, and hypertension*.

*Model^c^: Adjustment for demographics (age and sex), left ventricular ejection fraction, N-terminal pro-B-type natriuretic peptide, high-density lipoprotein cholesterol, estimated glomerular filtration rate, coronary artery disease, acute myocardial infarction, percutaneous coronary intervention, atrial fibrillation, diabetes mellitus, hypertension, spirolactone, angiotension-converting enzyme inhibitor or angiotension receptor blocker, and diuretic*.

### Predictors of Worsening LVEF

The mean LVEF of worsening patients with LVEF dropped from 65.1 ± 7.2 to 53.5 ± 10.5 within 3 to 12 months after discharge. In the multivariate binary logistic regression analysis [including LVEDD, LVEF, high-density lipoprotein cholesterol (HDL-C), NT-proBNP, AF, AMI, DM, beta-blocker, and angiotensin-converting enzyme inhibitor/angiotensin receptor blocker (ACEI/ARB)], LVEDD [odds ratio (OR): 1.05, 95% CI: 1.03–1.06], LVEF (OR: 1.14, 95% CI: 1.12–1.17), HDL-C (OR: 0.60, 95% CI: 0.38–0.94), AF (OR: 1.69, 95% CI: 1.28–2.25), and DM (OR: 1.51, 95% CI: 1.07–2.13) were detected as independent risk factors of worsening LVEF (Table 3).

**Table 3:** The univariate and multivariate logistic regression analysis of worsening LVEF.

	**Univariate**		**Multivariate**	
**Risk factors**	**OR (95% CI)**	***p*-value**	**OR (95% CI)**	***p*-value**
Age, years	1.00 (0.99–1.02)	0.725		
Female	1.05 (0.80–1.37)	0.715		
LVEDD, mm	1.05 (1.03–1.06)	<0.001	1.05 (1.03–1.06)	<0.001
LVEF, %	1.14 (1.12–1.17)	<0.001	1.14 (1.12–1.17)	<0.001
HDL-C, mmol/L	0.54 (0.33–0.87)	0.013	0.60 (0.38–0.94)	0.026
LDL-C, mmol/L	1.11 (0.96–1.29)	0.152		
NT-ProBNP, per 1,000 pg/ml	1.03 (1.00–1.06)	0.090	1.02 (0.99–1.05)	0.125
AF	1.77 (1.32–2.39)	<0.001	1.69 (1.28–2.25)	<0.001
AMI	1.27 (0.78–2.05)	0.337	1.50 (0.97–2.29)	0.064
Hypertension	1.06 (0.77–1.45)	0.734		
DM	1.43 (0.99–2.06)	0.054	1.51 (1.07–2.13)	0.018
CKD	0.86 (0.61–1.20)	0.368		
pre-PCI	1.64 (0.80–3.27)	0.166		
PCI	1.28 (0.80–2.04)	0.301		
B-blocker	1.20 (0.91–1.59)	0.190	1.25 (0.96–1.64)	0.105
Spirolactone	1.04 (0.62–1.79)	0.872		
ACEI/ARB	1.31 (0.90–1.90)	0.161	1.35 (0.95–1.93)	0.096
Diuretics	1.02 (0.58–1.79)	0.947		

*AMI, acute myocardial infarction; DM, diabetes mellitus; CKD, chronic kidney disease; AF, atrial fibrillation; PCI, percutaneous coronary intervention; LDL-C, low-density lipoprotein cholesterol; HDL-C, high-density lipoprotein cholesterol; NT-proBNP, N-terminal pro-B-type natriuretic peptide; LVEDD, left ventricular end-diastolic dimension; LVEF, left ventricle ejection fraction; ACEI, angiotension-converting enzyme inhibitor; ARB, angiotension receptor blocker*.

## Discussion

To the best of our knowledge, this is the first cohort study to examine the association of early worsening LVEF with mortality in patients with HFpEF and to establish independent predictors of worsening LVEF. The principal finding was that approximately one-third of patients with HFpEF would experience worsening LVEF, which was associated with a 2.2-fold increased risk of mortality. In addition, we found that increased LVEDD and LVEF, low HDL-C levels, AF, and DM were predictors of worsening LVEF.

In light of the present findings, the primary issue to be emphasized is the incidence of worsening LVEF. In this study of patients with HFpEF, 32.2% patients developed worsening LVEF, defined as an absolute decline in LVEF ≥ 5% from baseline 3 to 12 months after discharge. Analogously, Dunlay et al. reported that 38.5% of patients in a cohort of 559 patients in the USA had a decline in LVEF to < 50% in a 5-year follow-up after diagnosis of HFpEF (10). Likewise, after evaluating patient data from international registries, Savarese et al. reported that 39% of patients with HFpEF progress to LVEF < 50% (16). However, Park et al. reported that in a Korean cohort of 471 patients, only 9.6% of patients with HFpEF developed an LVEF < 50% at a 1-year follow-up (8). Similarly, Lupón et al. concluded that only 11.1% of 126 patients with HFpEF in Spain developed an LVEF < 50% during an 11-year follow-up, which suggested that most patients with HFpEF tend to maintain their HF prototype over time (9). Previous studies focusing on declining LVEF represented it as a “transition phenotype” leading to HFrEF, but in fact, only a small proportion of patients with HFpEF show such a decline. In this study, early, modest worsening LVEF occurred mainly in the population with an LVEF of 60 to 84% at baseline. The correlation between baseline LVEDD and baseline LVEF might seem a priori inconsistent. This may be one of the potential reasons why patients with an LVEF ≥ 70% are also prone to worsening EF. However, despite its importance, physicians are often unaware of the occurrence of early, modest worsening LVEF, which, thus, remains frequently unmanaged. Accordingly, it is necessary to regularly screen for early and modest worsening LVEF and explore the associated risk factors.

Few studies have comprehensively analyzed risk factors for worsening LVEF. In this study, higher baseline LVEDD is validated as an independent predictor of early worsening LVEF occurring 3 to 12 months after discharge. Recently, Abe et al. indicated that LVEDD was an independent predictor of recovered LVEF, defined as LVEF < 40% during hospitalization with progression to > 50% after a mean follow-up of 4 months (17). This proves finding supports the idea that LVEDD is related to LVEF trajectory in patients with HF. We also find it interesting that higher baseline LVEF is associated with an increased risk of worsening EF, which may be related to poor management of diet and exercise in these patients. On the other hand, a higher baseline HDL-C level is recognized as a protective factor for cardiovascular events (18). However, our results contrast with those of Karadag et al., who indicated that low HDL-C levels were not related to LVEF among patients with an LVEF ≤ 35% (19). However, the latter study should be interpreted with caution given its small sampling (*n* = 109) and the fact that it focused on high-risk patients (LVEF ≤ 35%). In addition, the presence of AF and DM are also potential predictors of worsening EF, which stresses for clinicians the need to pay attention to these indications and take the necessary precautions.

Another issue that should be remarked is the risk of mortality associated with early declining LVEF. In this study, worsening LVEF was associated with a 2.2-fold increased risk of mortality relative to nonworsening LVEF. Few studies have examined the prognostic impact of early worsening LVEF in patients with HF. Dunlay et al. reported that a 5% decline in LVEF during 5-year follow-up was associated with a 7% increased risk of mortality among patients with HFpEF (10). However, it is often difficult for clinicians to monitor LVEF variations for extended period. Kalogeropoulos et al. showed that patients whose LVEF transitioned from ≥ 40 to < 40% had subsequently a 5-fold higher mortality compared with those who maintained an LVEF ≥ 40% (20). Park et al. reported that a decline in LVEF to < 50% in patients with HFpEF was associated with an 82% increased risk of 4-year mortality (8). However, the results of the two aforementioned studies contrast with those of Lupón et al., who found, albeit in a small population sample, that a decline of LVEF to < 50% in patients with HFpEF was not associated with an increased mortality (9). One possible explanation for the poor prognosis of worsening LVEF is that “the term chronic stable HF is misleading, if cardiac structure and function remain deranged, even if symptoms have stabilized or no longer evident” (9). A likely contributor to the higher mortality of patients with HFpEF with LVEF decline is an heterogeneous pathophysiology, which often combines multiple comorbidities such as myocardial amyloidosis, hypertrophic cardiomyopathy, and cardiac sarcoidosis, making the diagnosis and treatment challenging (21). In addition, left ventricular longitudinal strain (LS) has emerged as a more accurate index to evaluate systolic function and predict prognosis (22). Both the exercise-induced B-lines assessing pulmonary congestion and the MEtabolic Road to DIAstolic Heart Failure (MEDIA) echocardiographic score were shown to independently predict prognosis and improve risk stratification in patients with HFpEF (23, 24). However, the latter were rarely measured in our cohort because of extra costs and infrequent application of these assessments in clinical practice. Thus, the evaluation of worsening LVEF seems to be best suited to the medical resources of developing countries. With these caveats in mind, further studies are clearly needed to clarify the relationship between worsening LVEF and mortality.

The 2020 American College of Cardiology/American Heart Association (ACC/AHA) guidance for HF emphasizes the management of HFrEF and fewer recommendations are provided in relation to HFpEF management (25). This study suggests that once diagnosed with HFpEF, patients should be re-evaluating LVEF at least annually. For these patients, a drop in LVEF ≥ 5% signals a danger warning that must be addressed by clinicians by adjusting treatment and management to avoid premature mortality. Overall, early diagnosis potentially optimizes the risk stratification of patients with HF with dynamic LVEF deterioration and provides elements to guide subsequent interventions of secondary prevention. Regarding treatment strategies, angiotensin receptor-neprilysin inhibitor (ARNI) and sodium glucose cotransporter-2 inhibitors (SGLT2i) have been listed as first-line medications by institutional guidelines and evidence-based medicine (26, 27). On the other hand, patients education on self-care practices and lifestyle advice are key measures to prevent the progression and improve the prognosis of HF. For instance, weight loss and exercise can improve LVEDD, cardiac activity, and quality of life to prevent worsening LVEF (28–31). Of note, nutritional supplementation of high-quality protein was suggested to prevent to some extent the progression of worsening LVEF (32).

## Limitations

Some limitations of this study should be considered. This was a single-center study, so its findings should be extrapolated cautiously to other settings. Although extensive adjustments were performed, we cannot rule out the influence of potential residual confounding factors. Besides, since LVEF remeasurements were clinically driven and different clinical events may alter the frequently of surveillance for LVEF, potential bias may have been introduced. We also acknowledge that a 5% decrease in EF is relatively small, but our results still suggested that early worsening LVEF is associated with a poorer prognosis. Moreover, we evaluated only all-cause mortality as the endpoint of the study and did not assess other dimensions of potential prognostic significance (e.g., readmission and quality of life). Therefore, larger prospective randomized controlled trials are needed to examine whether the application of treatment measurements targeting early LVEF declines will improve the prognosis of patients with HFpEF.

## Conclusion

This study indicated that approximately one out of three patients with HFpEF experiences worsening LVEF, which is associated with a 2.2-fold increased mortality. Increased LVEDD and LVEF, low HDL-C levels, AF, and DM were all the predictors of worsening LVEF in our patients cohort. While further studies are clearly needed to prospectively assess the efficacy of early active management on prognosis in patients with HF with worsening LVEF, physicians should pay close attention to signs of early worsening LVEF in patients with HFpEF and promptly implement early effective management.

## Data Availability Statement

The original contributions presented in the study are included in the article/supplementary material, further inquiries can be directed to the corresponding authors.

## Ethics Statement

The study conformed to the principles outlined in the Declaration of Helsinki and was approved by Guangdong Provincial People's Hospital institutional review board. Written informed consent for participation was not required for this study in accordance with the national legislation and the institutional requirements.

## Author Contributions

LiC, ZH, KC, LoC, and SC contributed to the research idea and study design. XZ, JLia, XL, YH, YK, YX, JLiu, JY, WY, WD, YP, JLu, YY, XX, and XQ contributed to the data acquisition. QX and KC contributed to the analysis and interpretation. ZH contributed to the statistical analysis. SC and KC contributed to the supervision and mentorship. SC and YL contributed to the writing guidance. All author contributed important intellectual content during manuscript drafting or revision and accepts accountability for the overall study by ensuring that questions on the accuracy or integrity of any portion of this study are appropriately investigated and resolved. All authors read and approved the final version of the manuscript.

## Funding

This study was funded and supported by the Beijing Lisheng Cardiovascular Health Foundation Pilot Fund (no. LHJJ20141751), the National Science Foundation of China (nos. 81970311 and 82070360), the study on the function and mechanism of the potential target for early warning of cardiorenal syndrome after acute myocardial infarction based on transformism (DFJH201919), and the Clinical Medicine Research Fund of Guangdong Province (2019ZX01). The funders had no role in the study design, data collection, and analysis, decision to publish, or preparation of the manuscript.

## Conflict of Interest

The authors declare that the research was conducted in the absence of any commercial or financial relationships that could be construed as a potential conflict of interest.

## Publisher's Note

All claims expressed in this article are solely those of the authors and do not necessarily represent those of their affiliated organizations, or those of the publisher, the editors and the reviewers. Any product that may be evaluated in this article, or claim that may be made by its manufacturer, is not guaranteed or endorsed by the publisher.
